# Social Expectations Bias Decision-Making in Uncertain Inter-Personal Situations

**DOI:** 10.1371/journal.pone.0015762

**Published:** 2011-02-09

**Authors:** María Ruz, Anna Moser, Kristin Webster

**Affiliations:** 1 Department of Experimental Psychology, University of Granada, Granada, Spain; 2 Department of Neuroscience, Brown University, Providence, Rhode Island, United States of America; French National Centre for Scientific Research, France

## Abstract

Understanding the role that social cues have on interpersonal choice, and their susceptibility to contextual effects, is of core importance to models of social decision-making. Language, on the other hand, is one of the main means of communication during social interactions in our culture. The present experiments tested whether positive and negative linguistic descriptions of alleged partners in a modified Ultimatum Game biased decisions made to the *same set of offers*, and whether the contextual uncertainty of the game modulated this biasing effect. The results showed that in an uncertain context, the same offers were accepted with higher probability when they were preceded by positive rather than by negative valenced trait-words. Participants also accepted fair offers with higher probability than unfair offers, but this effect did not interact with the valence of the social descriptive words. In addition, the speed of the decision was affected by valence: acceptance choices were faster when they followed a positive adjective, whereas rejection responses were faster after a negative-valenced word. However, these effects were highly reduced when the uncertainty was eliminated from the game. This suggests that positive and negative *relevant* social information can bias decisions made to the same pieces of evidence during interpersonal interactions, but that this mainly takes place when the uncertainty associated with the choices is high.

## Introduction

Making decisions is a common chore in our daily lives. From small-range choices, such as where to have dinner, to long-scale dilemmas such as whether to change jobs, we constantly find ourselves in situations in which we have to consider the available options and potential outcomes and their value, and choose according to our short and/or long-term goals. The nature of our decisions significantly influences our well-being and satisfaction, and deficits in decision-making may have disastrous consequences for our lives [1]. Given the extensive social nature of humans, many of our choices involve other people.

An important line of research considers which aspects of the current state of affairs modulate our decisions in social contexts. It is broadly accepted that deciders are not purely rational agents who only take into account self-interest, fixed preferences and objective information [2], [3], [4]. In many cases, subjective judgements about events are mediated by heuristics, which produce predictable biases [5]. Other biasing factors related to the deciders include the stereotypes they hold [6], their incidental emotional state [7], [8], or general tendencies to conform to the norms of the group [9]. In addition, people often rely on various social cues in their environment to guide their decisions. For example, social information about others, such as their moral status [10], incidental feelings [11] or displayed emotions influence decisions towards them.

Previous studies using economic games have shown that people gather social information about strangers to guide their choices as to whether or not to trust them. In situations in which people lack information regarding their partners, they may use social cues with positive or negative connotations to create a representation of the mind of the others [12] and use this to predict their most likely behaviour. For example, Delgado, Frank & Phelps [10] showed that partners described as morally praiseworthy were trusted more often than those described as having neutral or suspicious moral traits in an iterated Trust Game. Similar results have been found for faces displaying positive or negative emotions [13], [14]. In a related line of research, van 't Wout, Kahn, Sanfey AG & Aleman [8] showed that implicit trustworthiness ratings of facial photographs displaying neutral emotions are highly correlated with the amount of money that participants invest on their partners during a Trust Game. Overall, these results suggest that people use different types of explicit and implicit cues to evaluate their partners in interpersonal situations and to inform their choices. The more the social cues indicate that the partners may reciprocate their investment, the higher the likelihood that they will cooperate with them.

It is currently unexplored, however, how social cues are used to evaluate situations that have already taken place. That is, given the same objective behavior, would social cues pertaining to other people influence how we judge their behavior, and how we react to it? From a rational point of view, evaluations and choices (e.g. accepting or rejecting a monetary offer in an economic game) should not change depending on the information we have regarding the person with whom we are interacting. On the other hand, social information has a highly salient status, and seems to be rapidly and automatically processed [15]. Thus, choices made in interpersonal interactions may be different depending on the knowledge we have regarding our partner, even when the objective situation to be judged remains the same across conditions.

An important factor known to influence decision-making is the uncertainty associated with a judgment [16]. In uncertain situations, our knowledge about how actions lead to different outcomes is not perfect [17]. The level of uncertainty may not only change the choice strategies [18], but also the neural circuit engaged by the decision-making process [19], [20]. From a reinforcement learning perspective, the value of information increases with uncertainty [21]. For example, in highly-volatile or rapidly-changing contexts, new events have a larger effect in the decisions than in more stable contexts, in which uncertainty is lower [18]. This tendency is observed in other domains as well. For example, investors are more sensitive to market news and tips during periods of unstable stock prices than during stable epochs [22].

The role of uncertainty in social situations is not currently well understood. Although this variable is often mentioned as an important characteristic of many inter-personal situations [23], [24], its role in modulating the weight that different sources of social information have in interpersonal decision-making has not been explored. Previous research suggests that this variable may play an important role. In his social comparison theory, Festinger [25] proposed that in ambiguous situations in which there are no objective means to evaluate our opinions and abilities, people use other people as a valuable source of evidence. This line of research has shown that uncertainty leads to a stronger identification with social groups [26] and enhanced in-group bias [27], and may also generate higher conformity to group norms [28]. Thus, uncertainty seems to predispose people to be influenced. A different line of research, however, suggests that social influence takes place under several circumstances, in an ‘automatic’, even unconscious fashion [29], [30], [31] and leads to expectations about how others may act [32].

The goal of the present study was to evaluate the role of uncertainty in the way people acquire and use social information during interpersonal decision-making. We explored if prior social information biases decisions to the same set of offers made in interpersonal interactions, and whether the uncertainty of the context modulates this effect. For this, we devised an experimental protocol inspired by a well-known economic bargaining experimental setting, the Ultimatum Game [3]. In the original game, one player (the *proposer*) splits a certain amount of money into two sums, one for him and the other for another player, the *responder*. He, in turn, can either accept the offer (and thus they both win their respective amounts) or reject it (and both get nothing). Although a purely rational responder would accept every offer to make money [33], results show that low or unfair offers are rejected about half of the times [3]. In the modified game, participants always played the role of responder to divisions of money presented on a computer screen, which allowed measuring their choices and their speed. To increase the sensitivity of the task to the experimental manipulations, participants were required to make choices within a certain time limit.

In our current society, language is one of the main means of conveying information. It has been shown that verbally instructing someone about the association between stimuli and events can produce similar effects than personally experiencing or observing such associations [34]. Thus, to mimic the information that people may have about the personality of other people they interact with, in our study every offer was previously preceded, on a trial-by-trial basis, by trait-valenced adjectives (such as *bright* or *cruel*) that described the alleged partner for each trial. As trait words are a powerful means of social description, representing highly abstract characterizations that are easily generalized [35], it was hypothesised that the *same* offers would be accepted with a higher probability when they were preceded by positive rather than by negative descriptions of the partners. In addition, as participants were asked to make speeded responses, the valence of the words was expected to affect the speed of the choices in a bias-congruent manner. Experiment 1 corroborated these hypotheses, and Experiment 2 showed that the effects were not due to a response-preparation bias. Experiment 3 manipulated the ambiguity of the context and demonstrated that the influence of social information on choices was reduced, although not obliterated, when the uncertainty of the game was eliminated. Finally, Experiment 4 suggested that the social information needed to be attributed to the partners in the game for the bias to take place.

## Experiment 1

The current experiment manipulated the valence of adjectives describing the partners in the game (positive vs. negative), the type of offers that the partners made to the participants (fair vs. unfair) and the time that elapsed between the adjective describing the partner and the presentation of the offer (100 vs. 1300 ms).

### Methods

#### Participants

Eighteen native English-speakers students from the University of Oxford community participated in the experiment (10 females, 20 years on average), which was approved by the University of Oxford Research Ethics Committee. They all signed a consent form and received course credits and a chocolate token in exchange for their participation.

#### Procedure and Design

Sixty-four adjective words (4–8 letters) that could be used to describe a person were selected from the Affective Norms English Word database (ANEW) [36]. Half of the words had a positive valence (7.6 in average, SD = 0.5) and the other half had a negative valence (2.7 in average, SD = 0.6). Both groups were equated in number of letters (6 in average) and frequency of use (mean  =  28) [37]. These words are listed in Table 1.

**Table 1:** List of adjectives used in the task.

Word	Valence	Frequency	Word	Valence	Frequency
Admired	7.74	17	Angry	2.85	45
Adorable	7.81	3	Broken	3.05	63
Brave	7.15	24	Corrupt	3.32	8
Bright	7.50	87	Crude	3.12	15
Capable	7.16	66	Cruel	1.97	15
Cozy	7.39	1	Dirty	3.08	36
Cute	7.62	5	Disloyal	1.93	2
Devoted	7.41	51	Failure	1.70	89
Elated	7.45	3	Feeble	3.26	8
Elegant	7.43	14	Foul	2.81	4
Friendly	8.43	61	Guilty	2.63	29
Gentle	7.31	27	Hatred	1.98	20
Grateful	7.37	25	Helpless	2.20	21
Happy	8.21	98	Hostile	2.73	19
Honest	7.70	47	Immoral	3.50	5
Humane	6.89	5	Insane	2.85	13
Intimate	7.61	21	Lonely	2.17	25
Jolly	7.41	4	Lost	2.82	173
Joyful	8.22	1	Moody	3.20	5
Lively	7.20	26	Nervous	3.29	24
Loved	8.64	56	Resent	3.76	8
Loyal	7.55	18	Rigid	3.66	24
Lucky	8.17	21	Rude	2.50	6
Merry	7.90	8	Scared	2.78	21
Mighty	6.54	29	Selfish	2.42	8
Nice	6.55	75	Severe	3.20	39
Proud	8.03	50	Terrible	1.93	45
Radiant	6.73	8	Troubled	2.17	31
Secure	7.57	30	Unhappy	1.57	26
Terrific	8.16	5	Upset	2.00	14
Wealthy	7.70	12	Violent	2.29	33
Wise	7.52	36	Weary	3.79	17
**Avg (** ***std*** **)**	**7.57 (** ***0.5*** **)**	**28.97 (** ***26*** **)**		**2.70 (** ***0.6*** **)**	**27.84 (** ***32*** **)**

Upon arrival to the lab, participants were explained that they were going to play a modified version of the ‘Ultimatum Game’ with several different partners, represented by the computer. They were told that the offers that they were going to receive through the computer were made by participants in previous experiments. To stress the plausibility of this scenario, they completed a Social Value Orientation questionnaire [38], in which they were asked to split fictional sums of money themselves.

During the game task, participants played the role of the responder with several alleged partners, who were never the same, in a modified Ultimatum Game. Their goal was to accumulate more money than all of their partners together. If this was accomplished, they would win the game and would receive a chocolate token as a prize. Participants were told that their partner for each trial had received an initial amount of fictional British pounds (£; always an odd number) and had to split it into two amounts, one for each of them. This offer was displayed at the centre of the screen, in the form of two single-digit numbers separated by a slash symbol. These two numbers (from 1 to 9) were never the same, and the difference between them was either £1 (‘fair offer’) or £4 (‘unfair offer’; see Table 2). The role of the participant was to either accept or reject the offer for each trial by pressing the mouse buttons with their index and middle fingers of their dominant hand (button assignment was counterbalanced across participants). If the participant accepted the offer, one amount was added to his/her account and the other was added to the partners' account. If the offer was rejected instead, no money was added to any account. To speed-up responses, participants were told that their choices had to be faster than 1500 ms; otherwise the highest value would be added to the partners' account. In addition, they were told that, on every trial, before the offer they would see a trait adjective which represented personal characteristics of their partner for that trial, gathered from several questionnaires, and that these may or may not be related to the offer the partners made. These trait words had a positive valence in half of the trials, and a negative connotation in the remaining ones. In fact, the valence of the word did not predict the offer: both positive and negative adjectives were followed by offers with a small (£1) or large (£4) difference between the two numbers (50%).

**Table 2:** Offers presented during the game.

Fair		Unfair	
Left	Right	Left	Right
1	2	2	6
2	3	3	7
3	4	4	8
4	5	5	9
5	6	6	2
6	7	7	3
7	8	8	4
8	9	5	1
2	1	6	2
3	2	7	3
4	3	8	4
5	4	9	5
6	5	2	6
7	6	3	7
8	7	4	8
9	8	1	5
**5 (** ***2.4*** **)**	**5 (** ***2.4*** **)**	**5 (** ***2.4*** **)**	**5 (** ***2.4*** **)**

Experiment 1 tested the effect of the valence of personal trait-adjectives on choices in an uncertain context, in which participants did not have a complete knowledge of all the information relevant to win the game. This uncertainty was achieved by not informing participants as to which of the two numbers in the offer represented their share of the split; therefore they did not know how much money they and their partner would add if they decided to accept the offer. Participants were informed that they were entitled to the same amount of money as all of their partners together across the whole experiment, in the sense that half of the times the highest value would be for them and in the other half it would be for the partner, but they lacked this information on a trial-by-trial basis. We manipulated the valence of the personal adjective presented before each offer (positive or negative), the type of offer presented (fair or unfair), and the inter-stimulus (ISI) interval between the word and the offer (short, 100 ms, or long, 1300 ms). With this design we could explore whether the valence of the personal information we receive regarding a person with whom we are about to interact influences our decisions to accept or reject offers that are objectively the same for both valence conditions, as well as whether the fairness of the offer interacts or not with this bias. Also, the analysis of the speed of the decisions allowed us to investigate whether the personal information conveyed by the verbal descriptors prepared participants to make a decision in a valence-congruent fashion. In addition, the short and long ISI conditions offered information regarding the temporal course of these potential effects.

**Figure 1:**
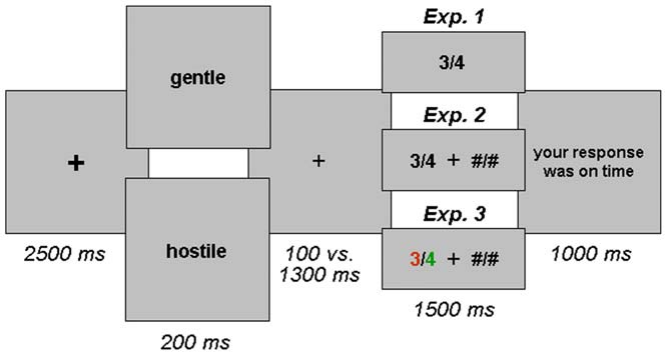
Schematic display of a trial sequence in Experiments 1, 2 and 3.

A PC running E-Prime software displayed the stimuli. Each trial comprised the following events (see Fig 1). A fixation point (+; 0.5°) was presented in the centre of the screen for a variable duration (1000–2000 ms) and then changed to bold font for another 1000 ms, which noticeably enlarged its size (0.6°). Then a positive or negative adjective (average 1.15°) was displayed in the same position for 200 ms. After an ISI of either 100 or 1300 ms, during which the central fixation point was presented, the offer (0.6°) was displayed in the centre of the screen for 1500 ms. Finally, a feedback message was displayed during 1000 ms. If the participants had responded within the 1500 ms window, it said “Response was on time”. If they did not respond on time, the message said, “The allowed response time has finished! Your partner adds the higher amount”. The following trial started immediately afterwards. Across all the experiments in this study, at the end of the task participants were informally debriefed about the game and their impressions about it. None of them reported suspicions regarding its rationale.

In total, participants received 256 offers, and the approximate duration of the game was 21 minutes. Before this, participants performed a short practice block to familiarize them with the task, in which faces of female and male partners (50%) displaying a neutral expression were presented instead of the trait-words.

The choices made by participants (% of accepted offers) were analyzed by a 2 (Offer: fair vs. unfair) X 2 (Valence: positive vs. negative) X 2 (ISI: 100 vs. 1300) multifactorial ANOVA. In addition, we explored the speed of choices, or decision times (DTs), in a 2 (Choice: accept vs. reject) X 2 (Offer: fair vs. unfair) X 2 (Valence: positive vs. negative) X 2 (ISI: 100 vs. 1300) multifactorial ANOVA.

### Results and Discussion

Participants responded on time in 98.3% of the trials and accepted 50% of the offers on average. The fairness of the offer influenced participants' choices: fair offers were accepted more often (M = 74%, SE = 25%) than unfair offers (M = 26%, SE = 16%), F_1,17_ = 26.80, p<0.001, η^2^ = .53. In addition, the valence of the adjectives also affected choices, with offers following positive words being accepted more times (M = 57%, SE = 11%) than offers followed by negative words (M = 43%, SE = 15%), F_1,17_ = 10.31, p = 0.005, η^2^ = .04. No other factors or interactions were significant (all ps>0.1).

**Figure 2:**
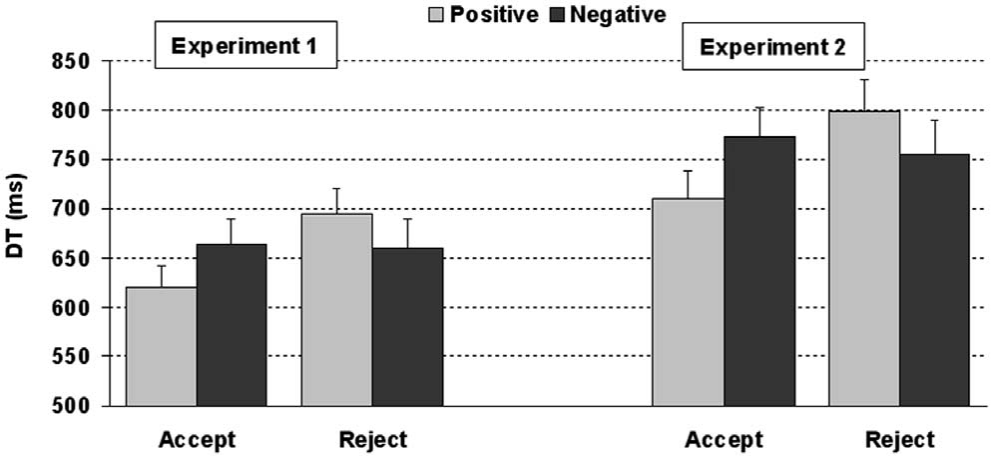
Decision times (in ms) for acceptance and rejection choices preceded by positive and negative trait-descriptive words in Experiments 1 and 2.

There was a main effect of ISI on DTs, F_1,17_ = 9.98, p = 0.01, η^2^ = .11, as responses were faster in the long (M = 636 ms, SE = 110) than in the short (M = 684 ms, SE = 112) ISI conditions. In addition, there was a significant interaction between the choice and the valence of the adjective F_1,17_ = 8.01, p = 0.01, η^2^ = .06. When participants accepted the offer, they did so faster when it was preceded by a positive (M = 620 ms, SE = 93) than by a negative (M = 665 ms, SE = 101) word, t(17) = −2.86, p<0.05. This effect was reversed when the offer was rejected, as responses were faster following a negative than a positive adjective (M = 660 ms, SE = 124, vs. 695 ms, SE = 108), t(17) =  2.47, p<0.05; see Figure 2.

The results of the present experiment, which used a modified version of the Ultimatum Game, show that both the type of offers (fair and unfair) and the valence of social information regarding the partners (positive and negative) have an independent influence in the decisions to the same set of offers made in an uncertain context. The major determinant of the choices made by participants was the fairness of the offer, as those in which the difference between the two amounts was small were accepted more often than those in which the difference was large. These results agree with several previous studies that have used the classic Ultimatum Game [3], which show that when the difference between the two amounts in the split is large, people have a higher tendency to reject the offer, even when this means that they will not make any money. Research using neuroimaging techniques has suggested that unfair offers generate negative emotions in the responders of the game, as evidenced by activations in the right anterior insula [39], which leads them to reject the offer.

In addition, and more importantly, our results showed that the valence of trait descriptive adjectives can bias choices *to the same set of offers* presented in an uncertain context. Participants were more likely to accept offers preceded by words that provided positive information about their partners than when these adjectives were negative in valence. Note that the lack of interaction between fairness and valence suggests that social information biased choices made to both fair and unfair offers in the same manner. In addition, DTs suggest that the polarity of the information provided by the personal adjectives somehow prepared participants to either accept or reject the offers. When the words were positive, participants accepted the offers faster than they rejected them, but rejection was faster when the descriptors were negative. Interestingly, none of these effects interacted with the ISI between the word and the offer, which suggests that the bias operates soon after the words are presented, and it remains effective for at least a second and a half.

As noted earlier, previous research had shown that personal social information influences the amount of trust that participants endow in their partners during economic games [8], [10]. In these cases, social cues are allegedly used to build a representation of the mind of the partners, to try to predict their future behavior [12]. The results of the present experiment complement the studies described above using a different approach. Participants played the role of *responders* in the game, that is, they had to make choices (accept or reject) regarding behaviors (the offers) *that had taken place already*. Participants were informed that the adjectives may or may not be related to the subsequent offer: in fact the offers were exactly the same for both conditions. Along the whole experiment, the valence of the descriptors did not predict whether the offer was going to be fair or unfair, as these two factors were manipulated in an orthogonal manner. Therefore, our results cannot be explained by any type of contingency learning taking place during the experiment, because there was no association between the valence of the words and the offers. In spite of this, the valence of the personal descriptors biased the choices that participants made to the same set of offers. The influence of this factor on the DTs suggests that participants used the words to prepare to make decisions that were consistent with the valence of the adjectives and this led to faster acceptance responses after positive words and faster rejection choices after negative descriptors.

From the results of this experiment, however, it is not clear whether words merely generated a response-related bias or whether they primed actual decision-making tendencies. That is, as the responses for accepting and rejecting the offers were fixed, participants may have merely activated a motor command upon presentation of the word. Instead of fully considering the offer, they may have made their decision based on the valence of the word, and prepared a motor response accordingly. Therefore, the effect of the words on the choices may be explained by a response-related bias or by the bias of decision-making options. Experiment 2 tested these ideas.

## Experiment 2

This experiment manipulated the same variables as Experiment 1 (valence of adjectives -positive vs. negative-, the type of offers -fair vs. unfair- and ISI -100 vs. 1300 ms-) in task slightly modified so participants could not prepare a motor response in advance.

### Methods

#### Participants

Eighteen native English-speakers students from the University of Oxford community participated in the experiment (9 females, 21 years on average), which was approved by the University of Oxford Research Ethics Committee. They all signed a consent form and received either course credits or £7 in exchange for their participation, plus a chocolate token.

#### Procedure and Design

The stimuli and procedure were the same as in Experiment 1, except in the following ways. To prevent the preparation of a motor response before the offers were presented, the offers were displayed to the left or right (50%) of the fixation point in an unpredictable manner. To balance visual stimulation across hemi-fields, two hash symbols separated by a slash were presented on the opposite side of the screen. Participants were instructed to press the mouse button of the side in which the two numbers were displayed on the computer screen if they wanted to accept the offer, and the opposite button if they wanted to reject it (see Figure 1).

### Results and Discussion

Participants responded on time in 98.8% of the trials and, on average, they accepted 47% of the offers. Similarly to Experiment 1, fair offers were accepted more often (M = 64%, SE = 25%) than unfair ones (M = 36%, SE = 25%), F_1,17_ = 7.22, p = 0.01, η^2^ = .2. In addition, offers that followed positive adjectives were accepted more often (M = 57%, SE = 19%) than those following negative descriptors (M = 43%, SE = 20%), F_1,17_ = 4.21, p = 0.05, η^2^ = .05.

DT were faster in the long (M = 727 ms, SE = 176) than in the short ISI (M = 775 ms, SE = 149), F_1,17_ = 5.31, p<0.05, η^2^ = .08. Also, the interaction between Choice and Valence was significant, F_1,17_ = 7.49, p<0.05, η^2^ = .06. In concordance with the previous experiment, responses accepting the offers were faster after positive (M = 710 ms, SE = 119) than after negative (M = 774 ms, SE = 126) words, t(17) = −3.331, p<0.01, whereas responses rejecting the offers were faster after negative (M = 755, SE = 149) than positive (M = 798, SE = 145) trait-descriptive adjectives, t(17) = 3.027, p<0.01 (see Figure 2).

In addition, we combined the results of Experiments 1 and 2 to test an additional hypothesis. If participants were actually attributing the meaning of the adjectives to their partners to guide their decisions, those words denoting characteristics closely linked to moral and trustworthy personalities should have a larger effect on acceptance rates than words without such associations. To test this, we selected the trials of Experiment 1 and 2 in which the meaning of the words had a close link to morality (positive: friendly, honest, humane, nice vs. negative: corrupt, selfish, disloyal, immoral), and those in which such relationship did not hold (positive: cozy, cute, elated, jolly vs. negative: lonely, lost, nervous, weary). First, we analyzed the acceptance rates with a 2 (Adjective content: moral vs. amoral) x 2 (Valence: positive vs. negative) x 2 (Offer: fair vs. unfair) multifactorial ANOVA. The interaction between the morality denoted by the adjective and its valence was highly significant, F_1,35_ = 12.26, p = 0.001. Whereas the valence of the moral adjectives heavily influenced decisions, F_1,35_ = 13.89, p<0.001, this variable did not have a significant effect for amoral adjectives, F_1,35_ = 1.85, p = 0.18. In addition, we explored the effect of the adjective content in the DTs by means of another ANOVA with the factors 2 (Adjective content: moral vs. amoral) x 2 (Valence: positive vs. negative) x 2 (Choice: accept vs. reject). Crucially, there was a third-order interaction between the morality of the adjectives, their valence and the choice, F_1,35_ = 4.65, p<0.05. Consistent with the previous results, the interaction between the choices and the valence of the adjectives when these were moral was significant, F_1,35_ = 15.38, p<0.001, whereas this interaction did not reach significance levels for amoral adjectives, F_1,35_ = 1.52, p = 0.22. Together, these results strongly suggest that the morality content of the words is highly relevant both for the decision to accept or not the offers and for the speed at which these decisions are made.

In this experiment, the unpredictability of the location of the offer prevented participants from preparing a motor response in advance. In spite of this, Experiment 2 replicated the results obtained in Experiment 1. Fair offers were more likely to be accepted than unfair ones, and the same happened for offers preceded by words with a positive valence compared with negative ones. In the same manner as in Experiment 1, there was no interaction between these two factors, which suggests that the role of valence was the same for both types of offers. However, valence had opposite effects for the two possible choices in the game, as positive words speeded acceptance responses and negative words made rejection decisions faster. That is, even when it is not possible to prepare a motor response upon presentation of the words, adjectives describing positive and negative qualities of partners in an economic game can influence choices and affect the speed of the responses in a decision-consistent manner. This suggests that information regarding other people rather than merely activating motor commands, primes decision-making tendencies and affects their speed when we make choices in uncertain situations. This is especially true for adjectives denoting personality characteristics related to morality.

An open question remains regarding the scope of these effects. The biasing effect of personal information may be restricted to uncertain social situations, in which participants have imperfect knowledge about the outcomes of their decisions, or it may extend to less ambiguous contexts, in which the potential outcomes of the different choices have been specified. In the present study, the lack of information regarding the assignments of the splits in the offers to the participant and the partner provides an ambiguity that may have predisposed participants to take into account the social information when making their decisions. Previous demonstrations of the effects of social cues on decision making [8], [13] have also used uncertain contexts, as participants had to play with unknown partners and did not have information regarding their behavior in previous interactions, except the social cues provided by the experimenters [40]. It may be that the uncertainty of the situation makes social cues valuable as the only available means to predict the behavior of the partner, and this is why they bias decisions. Another possibility is that social information is highly salient in any dynamic interaction between people and thus it may affect choices even in unambiguous contexts. The following experiment intended to contrast these two options by including a block of trials with high uncertainty (as in the previous experiments) and another block with low uncertainty. To reduce uncertainty, the two numbers in the offer were colored with different hues, and participants were informed which of the two colors would always add to their account in case they accepted the offer. Thus, in the certain block participants had a complete account of the consequences of their choices, which reduced the ambiguity present in the previous experiments.

## Experiment 3

This experiment manipulated the same variables as Experiment 1 and 2 (valence of adjectives -positive vs. negative-, type of offers -fair vs. unfair- and ISI -100 vs. 1300 ms-) and added the variable of contextual uncertainty (certain vs. uncertain block). In the uncertain block, the offers were presented in black (as in the previous experiments) and thus the uncertainty of the game was high. In the certain block, the two numbers in the offers were colored in different hues. This informed participants about the specific amount that they would add if they accepted the offer and thus reduced the uncertainty associated to the game.

### Methods

#### Participants

Twenty-six native Spanish-speakers students from the University of Granada participated in the experiment (22 females, 20 years on average), which was approved by the Ethics Committee of the Department of Experimental Psychology of the University of Granada (Spain). They all signed a consent form and received course credits in exchange for their participation.

#### Procedure and Design

As Experiment 3 was conducted in Granada (Spain), we made a new list composed of Spanish words. Stimuli were 64 adjectives from the Spanish translation of the ANEW database [41]. They were matched to the stimuli in the previous list in terms of number of letters, frequency, valence and arousal ratings (all the Fs comparing the two lists were <1).

Experiment 3 had two blocks (its order was counterbalanced across participants), which differed in the level of uncertainty of the game. In the uncertain block, the procedure was the same as in the previous experiments. The certain block differed in the color of the numbers that comprised the offer. One of them was red and the other one was green. The color assignment was balanced with respect to the magnitude of the numbers (i.e. the higher number was green in half of the trials) and their relative location in the offer (i.e. the number presented to the left was green in half of the trials). The uncertainty of the game in this block was reduced because the experimenter informed participants which of the two numbers would be added to their account if they accepted the offer. Half of the participants were assigned the amount coded in green, and the other half received the amount colored in red. To maintain the duration of the game comparable to the previous experiments, participants received the same number of offers (256).

The choices made by participants (% of accepted offers) were analyzed by a 2 (Block: certain vs. uncertain) X 2 (Offer: fair vs. unfair) X 2 (Valence: positive vs. negative) multifactorial ANOVA. In contrast to the previous experiments, we did not analyze the speed of the decisions. The introduction of a new variable reduced in half the number of trials per condition, which resulted in decreased power. This made the analysis of the DTs unreliable. These data, however, were not relevant for the hypothesis tested in Experiment 3. The practice block was the same as in the previous experiments.

### Results and Discussion

Participants responded on time in 98.2% of the trials. During the block with high certainty, they accepted 90% of the beneficial offers and only 10% of the non-beneficial type (all the participants “won” the game).

**Figure 3:**
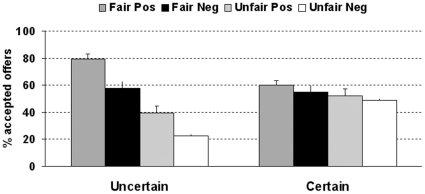
Acceptance rates for fair and unfair offers following positive and negative adjectives in uncertain and certain contexts in Experiment 3.

The certainty of the block had an effect on decisions: participants accepted more offers in the certain (M = 53.5%, SE = 10%) than in the uncertain (M = 49.7%, SE = 27%) context, F_1,25_ = 4.38, p<0.05, η^2^ = .01. The type of offer (fair or unfair) affected the decisions as well. Participants accepted more fair (M = 62.5%, SE = 17%) than unfair (M = 40,5%, SE = 17%) offers, F_1,25_ = 29,38, p<0.001, η^2^ = .24. The valence of the adjectives had an impact on the responses. Offers preceded by a positive adjective (M = 56.7%, SE = 13%) were more likely to be accepted than those preceded by a negative adjective (M = 46,3%, SE = 13%), F_1,25_ = 12,87, p<0.01, η^2^ = .05. In addition, there was an interaction between the type of the offer and the certainty of the context, F_1,25_ = 24,03, p<0.001, η^2^ = .1. The effect of the offer (i.e. fair minus unfair offer acceptance) was larger in the uncertain (36,7%; t(25) = 5.47, p<0.001) than in the certain condition (7,4%; t(25) = 2.87, p<0.01). Also, whereas fair offers were accepted more often in the uncertain than in the certain context (67.8% vs. 57.2%, t(25) = 3.1, p<0.01), unfair offers were accepted less often in the uncertain than in the certain context (31.1% vs. 49.8%, t(25) =  −4.8, p<0.001). Finally, there was an interaction between the certainty of the context and the valence of the words, F_1,25_ = 5,28, p<0.05, η^2^ = .02. In the uncertain condition, the effect of the valence (i.e. the acceptance rate after positive words minus the acceptance rate after negative words) of adjectives was higher (16,7%; t(25) = 3.25, p<0.01) than in the certain condition (4,4%; t(25) = 2.03, p = 0.05); see Figure 3.

In this experiment, the inclusion of a block with colored numbers as valid cues that reliably informed participants about the best choice to win the game (i.e. accept offers in which the higher number would be added to their account and reject those in which the partner would receive the higher amount) changed the pattern of results with respect to the previous experiments. Although the influence of fairness and social information in the choices was not completely obliterated in the block with low uncertainty, their effect was reduced compared to the high uncertainty context.

The fairness of the offers influenced decision-making once again: fair offers were accepted more often than unfair offers. Also, the type of offer interacted with the block: the effect of the fairness of the offer was larger in the uncertain than in the certain block. Intriguingly, whereas fair offers were accepted more often in the uncertain than in the certain context, this pattern was reversed for unfair offers, which were accepted less often in the uncertain than in the certain block. These differences may stem from the use of different strategies in the two contexts. Whereas the lack of information in the uncertain context may have led participants to rely heavily on the type of offer, the weight of this factor may have been toned down in the certain context given the availability of additional and game-relevant cues. Thus, the increase and decrease of acceptance rates driven by positive and negative words in the uncertain context may have been brought closer to mean acceptance rates (i.e. 50%) in the certain block, which could explain the differences. Note, however, that these results were not predicted and may need additional studies to be fully explained.

In addition, the valence of the adjectives had an effect on the acceptance rates, which also interacted with the type of block, as the effect of this variable was larger in the uncertain than in the certain block. Therefore, it seems that when the uncertainty of the situation is reduced, the fairness of the offer and also the social information provided by personal adjectives have a much smaller weight into the decision of accepting or rejecting the offers.

It is not clear, however, to what extent the *attribution* of personality characteristics to the partners was the crucial factor biasing the decisions. It could be argued that given that the nature of the task is rather artificial, in the uncertain block participants relied on the valence of the words because they had nothing else to go on. At the same time, the positive or negative valence of the words might have just caused *emotional priming* in the participants that biased them to accept offers related to positive words and to reject offers associated to negative words [7], [23]. To test this alternative explanation, we performed a final study. In Experiment 4, participants were told that the computer presented the words at random.

## Experiment 4

This experiment reproduced the variables manipulated in Experiment 3 (valence of the words, type of offer and uncertainty of the blocks), with the only variation being the instructions given to participants.

### Methods

#### Participants

Twenty-six native Spanish-speakers students from the University of Granada participated in the experiment (21 females, 21 years on average), which was approved by the Ethics Committee of the Department of Experimental Psychology of the University of Granada (Spain). They all signed a consent form and received course credits in exchange for their participation. Data from one participant was lost due to computer failure.

#### Procedure and Design

Apparatus and Stimuli were the same as in Experiment 3. Participants were told that before every offer, the computer would present a word at random that was unrelated to the task or to their partners. All the other details of the procedure were identical to the previous experiment.

### Results and Discussion

Participants responded on time in 97.6% of the trials. During the block with high certainty, they accepted 91% of the beneficial offers and only 18% of the non-beneficial type (as in the previous experiments, all the participants “won” the game).

The ANOVA showed a main effect of the type of offer, as fair offers (M = 68.7%, SE = 14.1%) were accepted more frequently than unfair offers (M = 39%, SE = 11.4%), F_1,24_ = 44.16, p<0.001, η^2^ = .37. In addition, there was an interaction between the certainty of the block and the type of offer, F_1,24_ = 40.87, p<0.001, η^2^ = .18, as the effect of the type of offer was larger in the uncertain (51%, t(24) = 7, p<0.001) than in the certain (9%, t(24) = 2.86, p<0.01) block. As predicted, however, the valence of the nouns did not have any effect or interacted with any other factor (all Fs<2.71, all ps>0.11).

Experiment 4 showed that when the words preceding the offers were not attributed to personality characteristics of the partners, they did not have any influence on participant's acceptance rates. As the adjectives used were the same as in previous experiments, this result suggests that the biasing effect that personality adjectives had on previous studies was not due to a mere emotional arousal generated by the words. It also suggests that in the uncertain context, participants did not use any kind of information available to guide their decisions, as if this were the case we would have observed a biasing effect also after irrelevant nouns.

## Discussion

The present study tested the idea that the personal information we have regarding other people with whom we interact may prepare us to take a line of action that is consistent with the valence of such prior social information, and bias the decisions we make in response to the same set of behaviors. Our results supported this conclusion, and further showed that this influence on decision-making is mainly present in uncertain contexts, in which participants lack precise information regarding the consequences of their choices.

Linguistic information is a powerful means to convey social information in our culture. When we lack first-person experience regarding a situation or another person, we often turn to information provided by others to form an opinion [25]. The present study showed that *non-predictive* personal information conveyed in the form of written verbal labels bias the choices we make to a set of offers that are objectively the same. Note that none of the experiments included any reliable association between the valence of the words and the nature of the offers. This allowed us to study the *spontaneous* bias that attributing personality characteristics to other people has on decision-making aside from experimental associations (e.g. between positive words and fair offers) that could have made such biasing an optimal decision strategy for participants.

Thus, it seems that the semantic content of the words ‘automatically’ prepared participants to make choices consistent with their valence, and thus acceptance choices were speeded after positive adjectives whereas rejection decisions were faster after negative-valenced words. The data, however, do not suggest that positive and negative words primed participants to expect fair or unfair offers, because we never observed an interaction between the valence of the words and the type of offer. The semantic information provided by the words may have focused the attention of participants in positive or negative personal characteristics of their partners, and this preparation may in turn have prepared them to accept the offers presented after positive words and to reject those appearing after negative personal information. This could then explain the speeding of the choices found for responses that were consistent with this attentional preparation. In addition, these effects took place with short and long intervals between the words and offers, which suggests that the biasing effect of social cues comes into operation soon after the information is presented, and remains active for at least a second and a half.

Further research would be needed to evaluate the level of processing at which this biasing effect takes place. Results from Experiment 2 ruled out a motor-preparation description, as results remained unchanged even when participants could not prepare a motor command upon presentation of the adjectives. One option is that the same offers were *perceived* or *evaluated* as more fair when they were preceded by words that described positive characteristics of the partner, compared to negative descriptions. More positive evaluations would lead to a higher acceptance rate, and this would be reflected in the results we obtained. Another possibility is that although offers were perceived in the same manner in both conditions, the adjectives biased the decision-making process, by priming acceptance responses after positive descriptors and rejection after negative ones. Future experiments employing neuroimaging techniques, which could offer information about the brain areas involved and/or the temporal profile of the effects, may shed some light on this respect.

The results of Experiment 3 suggested that the effect of the valenced-words on choices is not obligatory, but rather modulated by the uncertainty of the context in which decisions take place. From a reinforcement learning computational perspective, it has been proposed that contextual uncertainty arbitrates between the use of two dissociable brain control systems. The dorsal striatum would be involved in responses made in habitual, well-learned situations, in which uncertainty is low, whereas the prefrontal cortex would become engaged in contexts of high uncertainty [19], [20]. In these situations, agents seem to be more receptive to new information, which becomes more valuable and is given a larger weight in the decision-making process [16], [18]. The results of the present study fit nicely into this general framework by showing that uncertainty also modulates the influence of prior social information in interpersonal decision-making. The nature of the information, in addition, is also relevant in the sense that not all the information available is used to guide choices. As the results of Experiments 2 and 4 suggest, the pertinence of the information to the decision (i.e. the attribution of the information provided by the words to the partners in the game) modulated the extent of its influence. Moral-related adjectives had a large effect, whereas nouns presented at random had no effect whatsoever.

The reduced effect of personal adjectives on choices during the certain block in Experiment 3 need not to be in conflict with the large body of social cognition literature that shows that our behavior is in many occasions influenced by priming from social information even when we lack conscious awareness of the primes [29], nor with the line of research showing that words are may be processed in an ‘automatic’ fashion, regardless of task demands [42]. Taking the results of the first three experiments together, it could well be the case that the semantic information provided by the words was processed regardless of the context and the level of uncertainty may have modulated the weight that this social information received in the evaluation and/or choice process.

In contrast to the valence of the social information, the fairness of the offer had an effect in the choices made by participants along the four experiments. Several lines of research have shown that humans are not purely rational agents, but take into account how the outcomes of behaviors affect others and have concerns for norms of fairness [3]. The preference for fairness has been established in many different cultures, although it seems to be absent in our closest living relatives, chimpanzees [43]. In the current modified version of the Ultimatum Game, the fairness of the offer did not have a direct effect on how much money the participant would win, but on how much they would accumulate in order to win the game eventually. In spite of this, participants did take the fairness of the offer into account when making their decisions, and they accepted more fair than unfair offers.

The fairness of the offer, however, did not interact with the valence of the adjective across our experiments. Previous studies [7], [8] have shown that inducing emotional states in participants during the Ultimatum Game influences specifically choices made to unfair offers, in the sense that negative moods enhance rejection rates for unfair offers. This suggests that such rejection is mediated by a negative emotional reaction, which is also supported by neuroimaging evidence [39]. Positive and negative personal information in our game, on the other hand, modulated both fair and unfair offers to the same extent, which suggests that these two sources of information affect choices in an independent manner. Further studies which manipulate both the emotion of the participants and the information about their partners in the game would be needed to clarify the relation between these two sources of bias in interpersonal decision-making.

The ecological validity of our experiments is limited. Participants were not engaged in real, live two-person interactions and they did not have any previous experiences with the alleged people they were playing with. But these features, in turn, provided additional experimental control that helped to prove the basic phenomena of interest (as observed in both percentage of acceptance rates and speed of responses). Future research should be aimed at using procedures more ecologically valid that could be used to generalize the effects observed in the current study to more natural contexts.

In summary, our results show that the attribution of trait-descriptive words to the partners in a modified Ultimatum Game biases the decisions made to the same set of objective offers. In Experiments 1 and 2, the speed of these decisions was also modulated in a bias-congruent fashion. These effects were reduced, although not eliminated, when the uncertainty of the game was lowered. Overall, these results extend previous studies using economic games by showing that judgments we make about the *same* behavior may be influenced by the knowledge we have about the personal characteristics of others.

## References

[1] Damasio A (1994). Descartes' Error: Emotion, Reason, and the Human Brain..

[2] Fehr E, Camerer CF (2007). Social neuroeconomics: the neural circuitry of social preferences.. Trends Cogn Sci.

[3] Camerer CF (2003). Behavioral game theory: Experiments in strategic interaction..

[4] Sanfey AG (2007). Social decision-making: insights from game theory and neuroscience.. Science.

[5] Tversky A, Kahneman D (1974). Judgment under Uncertainty: Heuristics and Biases.. Science.

[6] Macrae CN, Stangor C, Hewstone M (1996). Stereotypes and stereotyping..

[7] Harle KM, Sanfey AG (2007). Incidental sadness biases social economic decisions in the Ultimatum Game.. Emotion.

[8] van 't Wout M, Kahn RS, Sanfey AG, Aleman A (2006). Affective state and decision-making in the Ultimatum Game.. Exp Brain Res.

[9] Asch SE, Guetzkow H (1951). Effects of group preassure upon the modification and distortion of judgments.. Groups, leadership, and men.

[10] Delgado MR, Frank RH, Phelps EA (2005). Perceptions of moral character modulate the neural systems of reward during the trust game.. Nat Neurosci.

[11] Andrade EB, Ho TH (2007). How is the boss's mood today? I want a raise.. Psychol Sci.

[12] Frith U, Frith CD (2006). How we predict what other people are going to do.. Brain Research.

[13] Scharlemann JPW, Eckel CC, Kacelnik A, Wilson RK (2001). The value of a smile: Game theory with a human face.. Journal of Economic Psychology.

[14] Ruz M, Tudela P (2011). Emotional conflict in interpersonal interactions.. Neuroimage.

[15] Willis J, Todorov A (2006). First impressions: making up your mind after a 100-ms exposure to a face.. Psychol Sci.

[16] Rushworth MF, Behrens TE (2008). Choice, uncertainty and value in prefrontal and cingulate cortex.. Nat Neurosci.

[17] Platt ML, Huettel SA (2008). Risky business: the neuroeconomics of decision making under uncertainty.. Nat Neurosci.

[18] Behrens TE, Woolrich MW, Walton ME, Rushworth MF (2007). Learning the value of information in an uncertain world.. Nat Neurosci.

[19] Daw ND, Niv Y, Dayan P (2005). Uncertainty-based competition between prefrontal and dorsolateral striatal systems for behavioral control.. Nat Neurosci.

[20] Hsu M, Bhatt M, Adolphs R, Tranel D, Camerer CF (2005). Neural systems responding to degrees of uncertainty in human decision-making.. Science.

[21] Dayan P, Kakade S, Montague PR (2000). Learning and selective attention.. Nat Neurosci.

[22] Schachter S, Hood D, Gerin W, Andreasson PB, Rennert M (1985). Some causes and consequences of dependence and independence in the stock market.. Journal of Economic Behavior and Organization.

[23] De Dreu CKW, Beersma B, Steinel W, Van Kleef GA, Kruglanski AW, Higgins ET (2007). The psychology of negotiation: Principles and basic processes.. Social psychology: Handbook of basic principles. 2nd ed.

[24] Van Kleef GA, De Dreu CKW, Manstead ASR (2010). An Interpersonal Approach to Emotion in Social Decision Making: The Emotions as Social Information Model.. Advances in Experimental Social Psychology.

[25] Festinger L (1954). A theory of social comparison processes.. Human Relations.

[26] Reid SA, Hogg MA (2005). Uncertainty reduction, self-enhancement, and ingroup identification.. Pers Soc Psychol Bull.

[27] Grieve P, Hogg MA (1999). Subjective uncertainty and intergroup discrimination in the minimal group situation.. Personality and Social Psychology Bulletin.

[28] Smith JR, Hogg MA, Martin R, Terry DJ (2007). Uncertainty and the influence of group norms in the attitude-behaviour relationship.. Br J Soc Psychol.

[29] Bargh JA, Chen M, Burrows L (1996). Automaticity of social behavior: direct effects of trait construct and stereotype-activation on action.. J Pers Soc Psychol.

[30] Bargh JA, Ferguson MJ (2000). Beyond behaviorism: on the automaticity of higher mental processes.. Psychol Bull.

[31] Higgins ET, Uleman JS, Bargh JA (1989). Knowledge accesibility and activation: Subjectivity and suffering from unconscious sources.. Unintended thought.

[32] Olson JM, Roese NJ, Zanna MP (1996). Expentancies; Higgins ET, Kruglanski AW, editors..

[33] Nash JF (1950). The bargaining problem.. Econometrica.

[34] Olsson A, Phelps EA (2004). Learned fear of “unseen” faces after Pavlovian, observational, and instructed fear.. Psychol Sci.

[35] Semin GR, Fiedler K, Stroebe W, Hewstone M (1991). The linguistic category model, its bases, applications, and range.. European Review of Social Psychology.

[36] Bradley MM, Lang PJ (1999). Affective norms for English words (ANEW): Instruction manual and affective ratings..

[37] Kucera, Francis WN (1967). Computational Analysis of Present-Day American English..

[38] Van Lange PA, Otten W, De Bruin EM, Joireman JA (1997). Development of prosocial, individualistic, and competitive orientations: theory and preliminary evidence.. J Pers Soc Psychol.

[39] Sanfey AG, Rilling JK, Aronson JA, Nystrom LE, Cohen JD (2003). The neural basis of economic decision-making in the Ultimatum Game.. Science.

[40] King-Casas B, Tomlin D, Anen C, Camerer CF, Quartz SR (2005). Getting to know you: reputation and trust in a two-person economic exchange.. Science.

[41] Redondo J, Fraga I, Padron I, Comesana M (2007). The Spanish adaptation of ANEW (affective norms for English words).. Behav Res Methods.

[42] Ruz M, Worden MS, Tudela P, McCandliss BD (2005). Inattentional amnesia to words in a high attentional load task.. Journal of Cognitive Neuroscience.

[43] Jensen K, Call J, Tomasello M (2007). Chimpanzees are rational maximizers in an ultimatum game.. Science.

